# Immunoediting and Antigen Loss: Overcoming the Achilles Heel of Immunotherapy with Antigen Non-Specific Therapies

**DOI:** 10.3389/fonc.2013.00197

**Published:** 2013-07-26

**Authors:** Arta Monir Monjazeb, Anthony E. Zamora, Steven K. Grossenbacher, Annie Mirsoian, Gail D. Sckisel, William J. Murphy

**Affiliations:** ^1^Department of Radiation Oncology, Comprehensive Cancer Center, University of California at Davis Medical Center, Sacramento, CA, USA; ^2^Department of Dermatology, University of California at Davis Medical Center, Sacramento, CA, USA; ^3^Division of Hematology Oncology, Department of Internal Medicine, Comprehensive Cancer Center, University of California at Davis Medical Center, Sacramento, CA, USA

**Keywords:** cancer immunotherapy, radiotherapy, immune tolerance, bystander T-cells, immune suppression, immune surveillance

## Abstract

Cancer immunotherapy has emerged as a mainstream therapy option in the battle against cancer. Pre-clinical data demonstrates the ability of immunotherapy to harness the immune system to fight disseminated malignancy. Clinical translation has failed to recapitulate the promising results of pre-clinical studies although there have been some successes. In this review we explore some of the short-comings of cancer immunotherapy that have limited successful clinical translation. We will give special consideration to what we consider the most formidable hurdle to successful cancer immunotherapy: tumor-induced immune suppression and immune escape. We will discuss the need for antigen-specific immune responses for successful immunotherapy but also consider the need for antigen specificity as an Achilles heel of immunotherapy given tumor heterogeneity, immune editing, and antigen loss. Finally, we will discuss how combinatorial strategies may overcome some of the pitfalls of antigen specificity and highlight recent studies from our lab which suggest that the induction of antigen non-specific immune responses may also produce robust anti-tumor effects and bypass the need for antigen specificity.

## Introduction

The allure of cancer immunotherapy as a potential magic bullet against cancer has intrigued researchers for over a century. The rationale underlying anti-cancer immunotherapy stems from the concept of immune surveillance first attributed to Erlich and colleagues over a century ago (1). This concept, founded in the idea that there is no evolutionary purpose to the tissue rejection immune response, states that tissue rejection is actually a manifestation of an immune surveillance mechanism that guards against spontaneously arising tumors. If such a mechanism does exist then it stands to reason that it can be re-invigorated and harnessed to battle malignancy in cancer patients. This idea, in its simplest form, is particularly attractive given that the immune system should be able to identify and specifically eradicate malignant cells based on the expression of abnormal antigens not expressed or present in normal tissues (2).

Interest in this concept has waxed and waned over the past century and during this time the major advances in cancer therapy were focused on cytotoxic therapies and surgical excision. Despite the continual advancement of the field, the inability to eradicate malignancy once it has disseminated remains the greatest challenge in cancer therapy. Over the past decade there has been a renaissance in cancer immunotherapy with a renewed belief by many that harnessing the immune system may be a viable strategy for successfully treating metastatic disease. This renaissance has produced a seemingly exponentially increasing number of pre-clinical and clinical studies which are serving to translate this concept into the mainstream arsenal of anti-cancer therapeutics. Numerous strategies are being explored including augmentation of antigen-presenting cells (APC) and immune effector cells, immunologic stimulants such as cytokines and pathogen associated molecular pattern (PAMP) receptor agonists, adoptive transfer of transgenic immune cells, antibodies and molecules such as anti-CTLA-4 antibody or transforming growth factor (TGF)-beta antisense aimed at reversing suppressive mechanisms, and numerous vaccines comprised of DNA, peptides, or autologous tumor cells [reviewed in Ref. (3, 4)]. Two such therapies, sipuleucel-T, a pulsed dendritic cell vaccine (5), and ipilimumab, an anti-CTLA-4 antibody (6), have been amongst the first to be approved by the FDA for mainstream use (although some immunotherapies such as Bacilli Calmette–Guerin have been used clinically for decades without the fanfare of the aforementioned therapies). These two therapies, which provide overall survival benefits in castration-resistant prostate cancer and melanoma respectively, have generated enthusiasm and played a central role in re-introducing immunotherapy into the mainstream. Unfortunately, the benefit imparted by these and other immune therapies remains modest. Sipuleucel-T provides no statistical benefit in freedom from progression and improves median overall survival by about 16 weeks (5). Similarly, ipilimumab also provides a survival benefit of roughly 16 weeks (6). Thus, although these therapies may validate the concept of cancer immunotherapy and are an important first step, they fall short of the theorized potential of eradicating metastatic disease. Unfortunately, to date, clinical studies of cancer immunotherapy have failed to manifest the pre-clinical and theoretical promise of this approach. In this manuscript we will review some of the hurdles of cancer immunotherapy including the need to overcome tumor-induced immune suppression and immune escape. We will discuss the importance of inducing antigen-specific immune responses for successful immunotherapy but also consider the need for antigen specificity as a major potential pitfall of immunotherapy given tumor heterogeneity, immune editing, and antigen loss. Finally, we will discuss how combinatorial strategies may overcome some of the pitfalls of antigen specificity and highlight recent studies from our lab which suggest that the induction of antigen non-specific immune responses may also produce robust anti-tumor effects and bypass the need for antigen specificity.

## Short-Comings of Cancer Immunotherapy

A recent publication summarizing results from the Society for Immunotherapy of Cancer (SITC) immunotherapy summit identifies nine critical hurdles in cancer immunotherapy (7). Of these nine critical hurdles, eight are related to the development of therapeutics and one is inherent to the therapies or diseases themselves. This one: the “complexity of cancer, tumor heterogeneity, and immune escape” encompasses a huge and diverse range of biological issues. Below we will consider some of these critical hurdles as well as other potentially critical obstacles.

One obstacle recently described by Lesterhuis and colleagues is that the timing and dosing for many immunotherapy regimens is often empirically derived and further refinement of these technical aspects may improve outcomes (8). In many ways this obstacle is directly attributable to the “limited funds available to translate science into patients.” The timing and dosing of many immunotherapy regimens tested in the clinic are extrapolated from pre-clinical regimens or from phase I trials assessing therapy tolerability and safety. Although the common thought with cytotoxic therapies is that the more that can be delivered, the greater the anti-tumor effect, this rationale may not hold true when trying to alter the delicate balance of the immune system for therapeutic gain. Issues such as exhaustion of effector cells and induction of suppressive networks must be considered.

Another critical hurdle is the “limitation of current animal models to predict the efficacy of cancer immunotherapy strategies in humans.” A recent publication has suggested, based on discordant gene expression profiles after traumatic or inflammatory insults, that mice provide poor models of human inflammatory diseases (9). It should be noted that a single mouse strain was used to draw such broad conclusions. Although this article has recently generated attention in the lay media, its conclusions regarding the short-comings of mouse models have long been recognized by most researchers. Despite these short-comings, mouse models remain a staple of pre-clinical studies due to the complex mechanistic studies which can be performed, low cost, and short generation times amongst numerous other advantages. These authors, while acknowledging the many limitations of mouse models, do not endorse abandoning a model which has over many decades proven its utility in improving the understanding and treatment of human disease. Care must be taken, however, to make our pre-clinical models as robust and accurate as possible and to properly validate pre-clinical findings prior to clinical translation. Most mouse cancer studies are performed in young mice, however human cancers most commonly occurs in the aged and the use of aged mice would be more appropriate for cancer studies. This is particularly relevant for immunotherapy studies given the significant changes in immune functioning with age. In a series of studies examining immunotherapy in young versus aged mice we have demonstrated a significant impact of age on efficacy and toxicity (submitted). We suggest use of aged mice should be considered as part of the pre-clinical development of any cancer study. We also suggest that companion animals with spontaneous tumors provide an excellent platform for validating pre-clinical studies prior to human translation.

Two other critical hurdles which we will consider together are “lack of definitive biomarkers for assessment of clinical efficacy of cancer immunotherapies” and that “conventional clinical response criteria do not take into consideration differences between response patterns to cytotoxic agents and immunotherapies.” Currently there is no reliable measure of treatment effects other than survival and imaging responses which makes it difficult to identify treatments that may have a small but important effect which needs to be further explored. The lack of validated assays that can measure immune response across trials make it difficult to determine how strategies should be altered to improve efficacy. These issues need to be explored at the pre-clinical level but also as correlative studies in clinical trials. Unfortunately, the capabilities of human immune monitoring fall short of the sophisticated assays used in pre-clinical models and further refinement and development are required (10). In many human trials immune monitoring correlatives consist of a simple characterization of various markers in the peripheral blood. Mouse (11) and human (12) studies demonstrate that the immune response observed systemically may not be representative of what is occurring in the suppressed tumor microenvironment and draining lymph nodes. Obviously, the ethical issues with justifying repeated biopsy of tumor or draining lymph nodes make this a dilemma, which is not easily resolved.

An issue not identified by the SITC summit is that clinical cancer trials of new agents are typically undertaken in patients with widely metastatic disease who, due to the large burden of disease, the immunosuppressive activities of the tumor itself, or the immunosuppressive effects of prior therapies, are unable to respond to even a very effective immunotherapy. Ohashi and colleagues have shown that anti-tumor vaccination is most effective after surgical de-bulking of the primary tumor (13) demonstrating that an effective immunotherapy alone may be unable to induce a clinically significant response if tumor doubling time is short or tumor burden is high. Not surprisingly, some of the greatest successes of immunotherapy have been produced in pre-invasive or very early stage cancers where there is a limited volume of disease and patients have received minimal prior therapy. For example, intravesicular *Bacille Calmette–Guerin* is a standard of care in the management of non-muscle invasive bladder cancer demonstrating superiority to chemotherapy in this setting (14) and an HPV peptide vaccine demonstrated a 50% complete response rate in women with pre-invasive vulvar neoplasia (15).

Another limitation of immunotherapy is the potential toxicity associated with many treatments. As we iatrogenically upset the delicate balance of the immune system we introduce the potential for severe adverse effects. Some therapies can produce systemic inflammation and cytokine storm with disastrous effects. The systemic administration interleukin (IL)-2 has demonstrated activity against metastatic renal cell carcinoma and melanoma capable of producing durable responses in patients with metastatic disease, but toxicities can be so extreme that it limits its regular use and treatments are often provided in intensive care units. In order to limit toxicity and mortality, such treatments are generally only undertaken at centers with expertise in IL-2 therapy but access to such centers is limited. Therapy can induce a severe vascular leak syndrome that emulates sepsis and is characterized by hypotension, vasodilation, pulmonary edema, neutrophil dysfunction, and, without intervention, culminates in end-organ failure and death (16, 17). In addition to a systemic inflammation and cytokine storm, another concern with immunotherapy is the induction of immune responses which inappropriately target self or through bystander effects damage self tissues. This autoimmunity is seen in certain instances in patients treated with ipilimumab, where therapy disrupts the immune suppressive mechanism network that prevents anti-cancer immune responses but also usefully prevents inappropriate immune responses. Disruption of the latter can produce autoimmune colitis, dermatitis, hepatitis, endocrinopathy, and other adverse effects (18). A report by Morgan et al. (19) illustrates the potentially disastrous effects of disrupting immune balance. T-Cells modified with chimeric antigen receptors (CARs) to HER-2/neu were transferred to a patient with refractory metastatic colorectal adenocarcinoma. Unfortunately, this patient suffered fatal pulmonary failure as the transfused T-cells unexpectedly recognized low levels of the HER-2/neu antigen present on lung epithelial cells. The adverse effects observed within this recent trial highlights the critical need to assess the short-comings of our pre-clinical models as a means to better foreshadow toxicity responses within the clinic.

**Figure 1:**
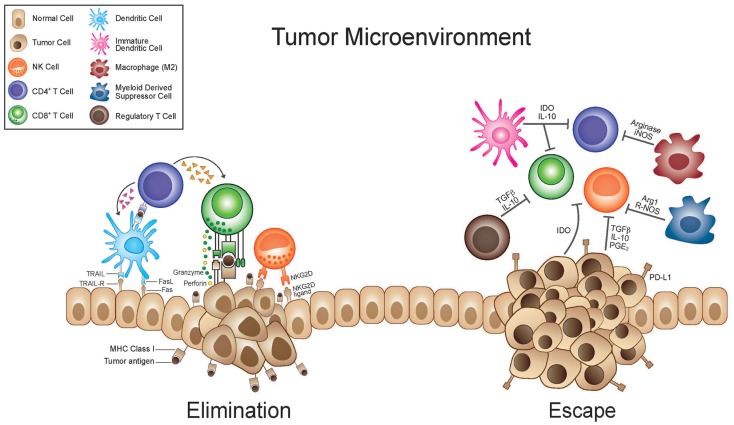
**Immunosuppressive tumor microenvironment and antigen loss mediate tumor escape**. During the elimination phase immune effector cells such as CTL’s and NK cells with the help of dendritic and CD4+ T-cells are able to recognize and eliminate tumor cells. This killing relies on stress ligands such as NKG2D and recognition of TAA’s in the TCR-MHC complex. As a result of tumor heterogeneity, tumor cells which are less immunogenic or have up-regulated immunosuppressive factors are selected for. These cells are able to subvert the immune response and escape immune surveillance. Tumor cells can secrete cytokines that recruit suppressive cells such as regulatory T (T_reg_) cells, immature myeloid cells [including immature dendritic cells (iDC) and myeloid-derived suppressor cells (MDSC)], and M2 macrophages. iDC can cause T-cell anergy due to lack of co-stimulatory molecules. M2 macrophages and MDSC inhibit T-cell responses through a variety of mechanisms, including nutrient sequestration via arginase, reactive oxygen species (ROS) generation, nitric oxide (NO), as well as interference with trafficking into the tumor site. Immunosuppressive cytokines and the up-regulation of immunosuppressive enzymes [like indolamine-2,3-dioxygenase (IDO) and arginase] that catabolize essential nutrients required for effector cell activation and also produce immunosuppressive catabolites, contribute to a microenvironment where immune responses are difficult to instigate and sustain. Furthermore tumor cells will down-regulate MHC molecules, loose expression of antigenic molecules, and up-regulate inhibitory molecules such as PD-L1.

Of the hurdles identified by SITC the “complexity of cancer, tumor heterogeneity, and immune escape” is the only one that addresses the nature of the disease itself. Under the umbrella of this one category fall a broad number of biological issues that are the subject of intense scientific investigation and will ultimately, more so than any of the other hurdles listed above, dictate the utility of anti-cancer immunotherapy. The “complexity of cancer” and “tumor heterogeneity” have been recognized for decades in mouse models. Fidler et al. demonstrated great variability in the metastatic potential of clones from a parent culture of murine melanoma (20). More recently, a genetic analysis of human renal cell cancers likewise demonstrated similar variability (21). Taking multiple spatially distinct biopsies from a single tumor the authors were able to demonstrate significant genetic changes within a given tumor providing evidence of the heterogeneity of even a single tumor. This topic and these studies will be considered in further detail later in this review. “Immune escape” can refer to a broad spectrum of mechanisms whereby an anti-tumor immune response is evaded or subverted. Two widely investigated phenomena that must be considered under this topic are immunoediting/antigen loss and tumor-induced immune suppression (Figure 1). Immunoediting is discussed in a separate section below and we will discuss the concept of tumor-induced immune suppression here (22, 23). The hostile nature of the tumor microenvironment and numerous mechanisms underlying this are well documented. The immune system is in a delicate balance of fluxes of activation and suppression that allow for appropriate responses but guard against potentially harmful responses that are inappropriate either in scale or target. A spectrum of suppressive cells, such as immature dendritic cells, regulatory T (T_reg_)-cells, myeloid-derived suppressor cells, and tumor-associated macrophages, are actively recruited to or generated within the tumor microenvironment (Figure 1). Likewise, a mélange of suppressive cytokines and enzymes, secreted by the tumor itself or resulting from the chronic inflammation associated with many tumors, contributes to the recruitment of the suppressive cells listed above and to direct suppression of effector cells. Cytokines such as TGF-beta, IL-10, and prostaglandin (PG)-E_2_ with documented immune suppressive effects may be highly expressed (24). Enzymes such as indolamine-2,3-dioxygenase (IDO) and arginase, which catabolize tryptophan and arginine respectively, can create a microenvironment in which immune effectors cannot activate or proliferate and suppressive cells thrive (25, 26). They function to both deplete the aforementioned amino acids essential for effector cell activity but also produce catabolites which can be independently suppressive and can alter the phenotype of immune cells from activating to suppressive (25, 26).

## Importance of Antigen-Specific Responses

The potential of cancer immunotherapy lies in the ability of the immune system to specifically distinguish and target non-self from self. Drawing on Erlichs earlier hypothesis, in the 1960s McFarlane Burnet and Lewis Thomas formally proposed the concept of immune surveillance as the true evolutionary purpose of the allograft tissue rejection response (27–29). They hypothesized that given the frequency of somatic mutations and that a proportion of these mutations will give rise to cells with malignant potential, then there must exist an evolutionary mechanism, likely immunological in nature, to deal with these potentially dangerous cells. This implies that tumors, although derived from host tissues must have some unique property whereby they can be distinguished from self. This concept of tumor antigens was confirmed in experimental animal models by Old and Boyse in the 1960s (2) who demonstrated the existence of tumor-specific antigens in murine leukemias and mammary tumors. These findings were later validated in human melanomas with the discovery of tumor-infiltrating lymphocytes able to recognize tumor antigens and lyse malignant cells (30). These findings have since been corroborated in various malignancies (31). The concept of tumor antigens has since evolved from tumor-specific antigens to tumor-associated antigens. These include inappropriately or over-expressed tissue antigens (i.e., Her-2/neu), viral oncogenes (i.e., v*-src*), idiotypic antigens (i.e., B-cell receptor), oncofetal antigens (i.e., CEA), fusion proteins (i.e., BCR-Abl), and post-translationally modified glycoproteins (i.e., MUC-1).

To date, the focal point of cancer immunotherapy research has been T-cell biology and by default tumor antigen-specific immune responses. A number of these therapies are being tested for clinical efficacy. As mentioned above, sipuleucel-T (Provenge), a pulsed dendritic cell vaccine, uses the prostatic acid phosphatase antigen, although the precise mechanism of its clinical benefit remains uncertain. Another strategy being tested is the use of CARs that engineer T-cells with receptors specific for target specific tumor antigens. Two clinical trials have demonstrated the potential of this approach. Infusion of T-cells with CARs targeting the CD19 B-cell antigen in chronic lymphoid leukemia (32) or the NY-ESO-1 antigen in synovial cell sarcoma and melanoma (33) has demonstrated the ability to induce tumor regression. It has been implicated that even therapies that are not necessarily billed as antigen-specific ultimately rely on the generation of antigen-specific T-cell responses for clinical effect. For example, clinical studies of CTLA-4 blockade with ipilimumab, which is aimed at reversing immune suppression, correlate clinical effect with the generation of T-cells specific to the NY-ESO-1 (34) and Melan-A (35) antigens. Similarly, Fong and colleagues demonstrate that clinical response of prostate cancer patients to ipilimumab is also correlated with the robustness of antigen-specific antibody and T-cell responses (36).

Given the existence of tumor antigens and their central importance in anti-cancer immunotherapy an approach being explored is the use of antigen-specific cancer vaccines. Unfortunately, these vaccinations, although successful in generating an antigen-specific immune response, have failed to produce meaningful clinical responses. For example, as announced by Therion Corporation by press release in 2006, a phase III trial of the viral PANVAC™ vaccine in pancreatic cancer patients elicited immune responses to the CEA antigen in about 70% of patients but without a survival benefit (http://www.prnewswire.com/news-releases/therion-reports-results-of-phase-3-panvac-vf-trial-and-announces-plans-for-company-sale-56997582.html). Moreover, Canvaxin™ – a melanoma vaccine – was able to induce antigen-specific responses to the glycoprotein tumor-associated antigen TA-90 (37), but Phase III trials were terminated early due to an observed survival detriment.

## Immunoediting and Immune Escape Subvert Antigen-Specific T-Cell Responses

One potential shortcoming of cancer immunotherapy not detailed above is the need for antigen-specific immune responses. Despite the findings chronicled above demonstrating the promise and importance of antigen-specific immune responses in cancer immunotherapy, many problems exist with this approach. One issue is the lack of clear target antigens for many tumors. Cancer arises from “self” tissues and thus the majority of antigens expressed have gained central tolerance. Few tumor-specific or tumor-associated antigens which can uniquely target malignant cells exist and those which do tend to be poorly immunogenic. This is in direct contrast to microbes that express a vast array of proteins, lipids, and carbohydrates which are foreign and strongly immunogenic. Nonetheless, some tumor antigens do exist and as demonstrated above immunity against them can be generated. The studies above also outline that even when a target antigen is identified and a response is generated, it may fail to translate into clinical benefit.

Although the reason for these findings is likely multi-factorial one of the most plausible explanations is that of immunoediting and antigen loss (Figure 1). As described by Schreiber and associates, this concept takes the principles of evolution and natural selection and applies them on a microscopic scale. It suggests that during carcinogenesis, tumors which become clinically relevant – under selective pressure by the host immune system – must have sub-populations which can survive immune pressure and are thereby selected for as the tumor evolves strategies to evade the host immune response (38). They describe three processes: the first is elimination during which active immune surveillance finds and eradicates the majority of tumor cells (or all of the tumor cells when it is successful). As a tumor grows and invades surrounding tissues the release of inflammatory cytokines recruit components of the innate immune system which will in turn, via cytokines and in the draining lymph nodes, recruit an adaptive immune response. The second is dynamic equilibrium during which time the rapidly dividing and mutating tumor is being eliminated by the immune system that is simultaneously placing an evolutionary pressure on the tumor and selecting out variants which by virtue of poor immunogenicity or other mechanisms are able to survive the immune attack. In the third phase, escape, tumor subclones which are poorly recognized or eliminated by the immune system are able to grow unchecked and become clinically observable disease. Experimental evidence from this same group demonstrates that tumors generated under selective immune pressure are less immunogenic (39). They find that chemically induced sarcomas from wild-type or RAG 2^−/−^ grow equally well when transplanted into naïve RAG 2^−/−^ mice but when transplanted into immunocompetent naïve wild-type mice less than half of the tumors generated in RAG 2^−/−^ grow as compared to 100% of those generated in wild-type mice. If tumors can become less immunogenic during carcinogenesis due to selective pressure from the immune system then it stands to reason that once established they can also evolve less immunogenic phenotypes if exposed to a new selective pressure from the immune system, as would occur with immunotherapy. Clinically, this concept is confirmed by the loss of the MART-1 antigen in melanoma patients after adoptive transfer of MART-1 specific T-cells (40, 41). In addition to antigen loss, there is evidence that tumor cells also down regulate the ability to present antigen, either by down-regulating major histocompatibility complex (MHC) or antigen processing capabilities (42, 43). Also supporting this hypothesis, it has been demonstrated that the patients who respond to antigen-specific therapies, such as a MUC-1 peptide pulsed dendritic cell vaccine, are those who have epitope spreading where an immune response is generated against tumor antigens not targeted by the antigen-specific therapy (44). This may be one mechanism whereby antigen-specific therapies can overcome this shortcoming and it may be that only those patients who are able to overcome the outgrowth of tumor subclones which poorly express MUC-1 are able to produce a clinically meaningful response, although this is not conclusively demonstrated.

**Figure 2:**
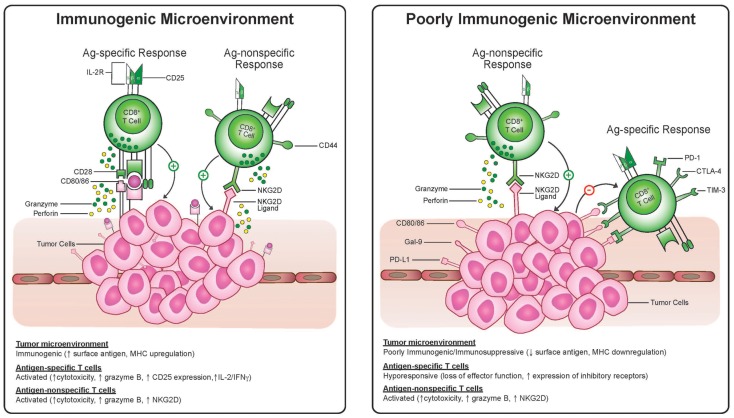
**Antigen-specific cytotoxic T-lymphocytes and antigen-non-specific bystander T-cell killing in an immunogenic and suppressive tumor microenvironment**. T-cells activated via TCR engagement up-regulate markers, including CD25, CTLA-4, and PD-1. Antigen-non-specific activated cytotoxic T-cells have a CD25^−^ and NKG2D^+^ phenotype. In the immunosuppressive environment antigen-non-specific activated T-cells may be resistant to suppressive signaling via PD-1 or CTLA-4 and may recognize targets expressing NKG2D ligands even when antigen is lost and MHC is down-regulated whereas antigen-specific T-cells may become anergic.

In this sense, tumor heterogeneity is a major obstacle in that the tumor subclones which do not express a given antigen or have some other trait which makes them poorly immunogenic will be selected for after immunotherapy. Recent genetic studies demonstrate the complexity of somatic mutations within a single melanoma (45) and the heterogeneity of spatially distinct biopsies from renal cell cancers (21) leading the authors of the latter to conclude that “intratumor heterogeneity, associated with heterogeneous protein function, may foster tumor adaptation and therapeutic failure through Darwinian selection.” As mentioned above, part of this process may entail selection of cells which are poorly immunogenic by virtue of low expression of tumor antigens or dysfunctional antigen-presenting machinery and part of this process may entail selection of cells which induce, by any number of mechanisms, an immunosuppressive tumor microenvironment (Figure 1). Antigen-specific effector T-cells appear to be particularly prone to both of these mechanisms since they rely on antigen recognition and are also more sensitive to direct suppression in the microenvironment, given up-regulation of molecules like PD-1, CTLA-4, Fas, and Lag-3 on antigen-specific activated T-cells (Figure 2). We propose that this may be an Achilles heel of many current immunotherapy approaches that limits both the magnitude and frequency of responses.

## Antigen Non-Specific Immunotherapy Approaches

There are some therapies that tend to be less susceptible to the short-comings of the antigen-specific therapies listed above. These types of therapies can include cytokines such as IL-2, immunostimulatory agents such as bacterial DNA, agonists and antagonists of key immunoregulatory molecules such as CD40 or PD-1, inhibitors of key enzymes such as cyclo-oxygenase or IDO, and vaccine strategies capable of encompassing the broad array and heterogeneity of tumor antigens such as syngeneic whole cell vaccines and *in situ* vaccines. As mentioned above in the discussion of ipilimumab, these therapies also rely, at least in part, on the generation of antigen-specific responses but we classify them for the purpose of discussion as antigen non-specific when they are not specifically targeted to one or a few antigens. They tend to be multi-modal and can have direct anti-tumor effects, target the suppressive tumor microenvironment, and activate innate immunity and adaptive immunity for both antigen-specific and antigen-non-specific killing (i.e., non-MHC-restricted killing by natural killer cells, macrophages, and T-cells) with most functioning through parallel mechanisms. For example, inhibition of cyclo-oxygenase can have direct cytotoxic effects on tumor cells by depriving them of necessary growth signals or inducing the intracellular accumulation of arachidonic acid (46, 47) and can also reverse immune suppression by blocking production of PGE_2_ (48, 49). Similarly, CpG oligodeoxynucleotides, which are recognized as bacterial DNA products and signal through toll-like receptor-9 (50, 51), can function to activate B-cells, dendritic cells, natural killer cells, macrophages, and lymphocytes but can also inhibit immunosuppressive myeloid-derived suppressor cells (52). Numerous approaches have been developed to target the immunosuppressive tumor microenvironment. Regulatory T (T_reg_)-cells are a well-studied component of tumor-induced suppression and can inhibit the function of effector T-cells and APC (53). The CD25 molecule (high affinity IL-2 receptor) expressed on T_reg_ cells is targeted by the antibody daclizumab (Zenapax) and by the IL-2 diphtheria toxin fusion protein denileukin diftitox (Ontak).

Activated T-cells up-regulate expression of the inhibitory CTLA-4 molecule and it competes with the co-stimulatory molecule CD28 for binding of B7, acting as a feedback inhibitory mechanism. This feedback inhibitory mechanism is taken advantage of by tumors that use it to inactivate effector T-cells in the tumor microenvironment. Blockade of CTLA-4 with ipilimumab has demonstrated promising clinical results with regression of melanoma in some patients and a benefit in median overall survival of 2.1 and 3.6 months in two clinical trials (6, 54). These findings validate the central role of CTLA-4 in maintaining tumor immune tolerance and suppressing tumor reactive T-cells.

Another strategy being tested in the clinical trials is inhibition of the immunosuppressive enzyme IDO. IDO is an inducible tryptophan-catabolizing enzyme which can function to induce tolerance to alloantigens as demonstrated by its prevention of T-cell-mediated fetal rejection in mice (26, 55). A growing body of evidence suggests that, akin to many other immunoregulatory mechanisms, IDO is high-jacked by tumors to induce tolerance and may even act as a master switch coordinating the different aspects of the suppressive tumor microenvironment. IDO has been demonstrated to be inappropriately expressed by tumors and can coordinate the induction of Tregs and inhibition of natural killer cells and effector T-cells and can be up-regulated to counteract the effects of cancer immunotherapy (56, 57). The potential of inhibiting IDO as a means to reverse tumor-induced immune suppression and promote an anti-tumor immune response has been demonstrated repeatedly in pre-clinical studies and both competitive inhibitors and small molecule inhibitors of this enzyme are being tested in clinical trials.

Massive expansion, activation, and non-MHC-restricted killing by NK cells, macrophages, and memory T-cells can be induced by intense immune-stimulatory therapies such as CD40 agonists, IL-2, GM-CSF, IL-12, and CpG. The advantage of these approaches is that they provoke a broad immune response involving many cell types and are not dependent on a specific antigen. As mentioned above, CpGs can activate APC such as dendritic cells through toll receptor-9 signaling (50, 58) causing increased antigen presentation, co-stimulation, and pro-inflammatory cytokine secretion which can, in turn, trigger innate and adaptive cell-mediated immunity (51).

Immunotherapy with potent cytokines such as IL-2 or IL-12 can provoke tumor rejection at least partially through a non-MHC-restricted mechanism that includes NK and T-cells (59–62). These cells likely identify and lyse target malignant cells by recognition of NKG2D ligands as opposed to specific antigens. Moreover, it has been demonstrated that IL-2 can cause conversion of NK and T-cells to lymphokine-activated killers which also recognize and kill target cells through an antigen independent mechanism (63). A recent report demonstrates the ability of a CD40 agonist to induce tumor regression of pancreatic adenocarcinoma in both a pre-clinical trial and a human clinical trial (64). The authors conclude that the anti-tumor effects are mediated by the cytotoxic effects of macrophages without a dependence on antigen-specific T-cells. These examples and the innumerable other such studies demonstrate the potential of this type of therapy to induce anti-tumor immunity. The advantage, at least in theory, is that they are not dependent on a specific cell type or antigen but instead create an environment where an anti-tumor immune response is supported and can efficiently recognize and eradicate malignant cells thus potentially rendering these therapies more resistant to the immune evasive mechanisms of tumors described above.

## Antigen Non-Specific T-Cell Responses

Classically, T-cells require two signals via the T-cell receptor (TCR) and a co-stimulatory signal for activation and proliferation, and the need for a third signal (cytokine secretion) has been described for cytotoxic effector function of CD8+ T-cells. These requirements for activation can, however, be bypassed in a phenomenon termed “bystander activation.” In the setting of very high cytokine stimulation, as occurs locally during viral and bacterial infections, memory T-cells can become activated and proliferate without the need for antigen-specific TCR engagement (65, 66). The exact function of these bystander activated T-cells is uncertain but in light of the up-regulation of cell surface NKG2D it has been suggested that they play a role in viral clearance (67, 68). Recently studies from our lab have demonstrated that bystander activated T-cells also occur after highly stimulatory systemic immunotherapy regimens (62) in a process that is similar to what is observed after infections such as influenza. These highly stimulatory immunotherapy regimens such as CD40 agonist and IL-2, CpG and IL-15, or IL-2 and IL-12 induce marked expansion of CD8^+^ T-cell compartment that primarily consists of CD44^high^ memory CD8^+^ T-cells (62). In murine models, the bystander memory CD8^+^ T-cells induced by these therapies proliferate and exhibit effector functions without the need for TCR engagement and produce significant anti-tumor effects which are dependent on IFN-g, IL-12, and Fas ligand expression but independent of CD4^+^ T-cells, NK cells, and perforin (69, 70). After immunotherapy (62) these antigen-non-specific activated memory CD8^+^ T-cells (AN-CTL) have a surface marker phenotype that is different than that of antigen-specifically activated T-cells through TCR engagement (Figure 2). In contrast to traditionally activated naïve or memory CD8^+^ T-cells, these AN-CTLs do not up-regulate surface expression of CD25 and PD-1 but do express the natural killer cell activating receptor, NKG2D giving them a unique NKG2D^+^CD25^−^CD8^+^ phenotype. The anti-tumor effects of these cells *in vivo* appears to be, in addition to IFN-g, IL-12, and Fas ligand, dependent on NKG2D as *in vivo* blockade of NKG2D significantly reduces the anti-tumor efficacy (62). These AN-CTLs express granzyme B and are post-therapy the only T-cells with cytolytic activity.

In TCR transgenic OT-1 mice, in which greater than 95% of the T-cells have TCRs specific for ovalbumin (OVA), vaccination with OVA produced OT-1 CD8^+^ T-cells which were able to lyse OVA-expressing EG7 tumor cells but not the OVA-negative EL4 parental cell line. Conversely, after highly stimulatory systemic immunotherapy with CD40 agonist and IL-2, bystander activated antigen non-specific OT-1 CD8^+^ T-cells are able to lyse both the OVA-expressing and OVA-negative targets *ex vivo* demonstrating their ability to kill without TCR engagement (62). Mirroring these results, *in vivo* therapy in OT-1 mice led to significant anti-tumor effects against OVA-negative 3LL tumors. We observed expansion of CD8^+^ T-cells expressing the unique phenotype of up-regulation of NKG2D and Granzyme B without up-regulation of CD25 or PD-1. Importantly, it appears a similar mechanism may exist in humans. Unlike cells activated by TCR engagement, *in vitro* IL-2-treated human memory CD8^+^ T-cells do not up-regulate PD-1 and CD25 expressing a similar bystander phenotype to that seen in mice. Furthermore, in melanoma patients, treatment with the topical toll-like receptor 7 agonist, imiquimod, produces infiltration of CD8^+^CD25^−^ T-cells compared to placebo-treated tumors (62). As a whole, these studies demonstrate that after highly stimulatory immunotherapy a pool of memory CD8^+^ T-cells expands and has effector function which is both independent of antigen-specific TCR engagement and plays a critical part in the anti-tumor efficacy of these therapies.

The anti-tumor effects of these AN-CTL have several advantages over traditional anti-tumor cytotoxic T-cells. Given that these cells are both activated and recognize their targets in an antigen non-specific manner they are less sensitive to the mitigating effects of immunoediting, MHC down-regulation, or antigen loss. As substantiated in the above studies using OT-1 mice bearing non-OVA-expressing tumors, these cells can exhibit anti-tumor effects even when a tumor antigen recognized by their TCR is lacking. Additionally, these cells may be less prone to immune suppression as the lack of PD-1 surface expression implies that they are impervious to suppression by PD-1 ligand expression on tumor cells. Furthermore, since these cells are derived from the memory compartment, they have presumably been through multiple rounds of selection (central and peripheral tolerance) and have shown the ability to recognize foreign antigens thereby deeming them “safer” to become activated in a non-specific fashion without causing autoimmunity. Clinically, another advantage of being derived from the memory T-cell compartment, is that memory T cells increase with age. Since most malignancies occur in an aged population with limited thymic output and a limited naïve T-cell compartment, these memory cells provide an attractive pool for immunotherapy. The disadvantage of this approach is the need for a cytokine rich environment; because they are lacking in CD25, the high affinity IL-2 receptor, these cells rely on copious amounts of cytokine to maintain their activated state which, as discussed earlier, has the potential to become extremely toxic.

## Overcoming the Achilles Heel of Immunotherapy with Combinatorial Strategies

It is likely that any successful immunotherapy strategy will need to rely to some extent on adaptive immunity, as any sustained response will need to rely on the development of immunological memory. This is demonstrated by the studies cited above of antigen-specific responses being correlated with outcomes in patients treated with ipilimumab. To date, the success of antigen-specific and non-specific strategies used alone have been unremarkable. Thus to overcome this Achilles heel of antigen-specific responses, yet still induce sustainable and clinically meaningful responses, will likely require the employment of combinatorial strategies using antigen-specific and non-specific approaches. This idea of targeting numerous mechanisms simultaneously to prevent the evolution of subclones which can circumvent the therapy has been highly successful in the management of HIV infection. For example, antigen-specific CD8 T-cells require TCR engagement whereas AN-CTLs recognize their targets via NKG2D ligands, thus the mechanisms of killing may be complimentary and a tumor would have to evolve strategies to overcome both of these mechanisms for immune evasion. Combinatorial strategies may also avoid some of the pitfalls of antigen-specific therapies and provide superior outcomes. As we have previously reviewed, the use antigen non-specific therapies can induce innate immunity and AN-CTLs that can “de-bulk tumors, increase antigen release, sway the tumor microenvironment from suppressive to permissive, and induce a milieu of pro-inflammatory cytokines” all of which serve to create an environment where antigen-specific therapies can be more effective (71). The idea that combining an antigen-specific therapy may be improved by combining with an antigen non-specific therapy has been demonstrated in a melanoma vaccine trial (72). Vaccinated patients produced a measurable antigen-specific T-cell response to the vaccine antigens but these vaccine primed responses were significantly increased following CTLA-4 blockade with ipilimumab. A similar question is whether these combinatorial strategies can not only increase the robustness of the response to the targeted antigens but induce a response against new tumor antigens. In a clinical trial using nodal injection of CpG molecules in melanoma patients, a response was generated against melanoma associated antigens in 50% of the patients suggesting that this approach could increase the antigens targeted after an antigen-specific therapy (73). Clinically, there is some data that these types of combination therapies may be an effective treatment strategy as the combination of immunomodulatory cytokines such as GM-CSF or IL-12 with vaccines has shown efficacy in preliminary trials (74, 75).

## Conclusion

Over the last decade cancer immunotherapy has evolved from a marginal idea to a reality in cancer therapy. Staggering breakthroughs are occurring with regularity and promising novel therapies that have the potential to change the paradigm of cancer treatment are on the horizon. Despite this promise and optimism the clinical efficacy of cancer immunotherapy has been modest to date. We have outlined above a number of potential obstacles to improving the effectiveness of immunotherapy. Chief amongst these is the need to overcome the tumors ability to evade an effective immune response. We suggest that combination immunotherapy regimens, working by many mechanisms simultaneously, may help address this obstacle. One approach which may be particularly useful in this regard is the induction of AN-CTLs which can bypass the need for antigen specificity and thereby help overcome the Achilles heel of cancer immunotherapy. Understanding how best to combine immunotherapy strategies is an area of active investigation which will further solidify immunotherapy in the arsenal of cancer therapeutics.

## Conflict of Interest Statement

The authors declare that the research was conducted in the absence of any commercial or financial relationships that could be construed as a potential conflict of interest.
